# VGLUT1 or VGLUT2 mRNA-positive neurons in spinal trigeminal nucleus provide collateral projections to both the thalamus and the parabrachial nucleus in rats

**DOI:** 10.1186/s13041-018-0362-y

**Published:** 2018-04-12

**Authors:** Chun-Kui Zhang, Zhi-Hong Li, Yu Qiao, Ting Zhang, Ya-Cheng Lu, Tao Chen, Yu-Lin Dong, Yun-Qing Li, Jin-Lian Li

**Affiliations:** 1 Department of Anatomy and K.K. Leung Brain Research Centre, The Fourth Military Medical University, Xi’an, People’s Republic of China; 2 Student Brigade, Fourth Military Medical University, Xi’an, People’s Republic of China

**Keywords:** Vesicular glutamate transporters, Spinal trigeminal nucleus, Thalamus, Parabrachial nucleus, Collateral projection, Rat

## Abstract

The trigemino-thalamic (T-T) and trigemino-parabrachial (T-P) pathways are strongly implicated in the sensory-discriminative and affective/emotional aspects of orofacial pain, respectively. These T-T and T-P projection fibers originate from the spinal trigeminal nucleus (Vsp). We previously determined that many vesicular glutamate transporter (VGLUT1 and/or VGLUT2) mRNA-positive neurons were distributed in the Vsp of the adult rat, and most of these neurons sent their axons to the thalamus or cerebellum. However, whether VGLUT1 or VGLUT2 mRNA-positive projection neurons exist that send their axons to both the thalamus and the parabrachial nucleus (PBN) has not been reported. Thus, in the present study, dual retrograde tract tracing was used in combination with fluorescence in situ hybridization (FISH) for VGLUT1 or VGLUT2 mRNA to identify the existence of VGLUT1 or VGLUT2 mRNA neurons that send collateral projections to both the thalamus and the PBN. Neurons in the Vsp that send collateral projections to both the thalamus and the PBN were mainly VGLUT2 mRNA-positive, with a proportion of 90.3%, 93.0% and 85.4% in the oral (Vo), interpolar (Vi) and caudal (Vc) subnucleus of the Vsp, respectively. Moreover, approximately 34.0% of the collateral projection neurons in the Vc showed Fos immunopositivity after injection of formalin into the lip, and parts of calcitonin gene-related peptide (CGRP)-immunopositive axonal varicosities were in direct contact with the Vc collateral projection neurons. These results indicate that most collateral projection neurons in the Vsp, particularly in the Vc, which express mainly VGLUT2, may relay orofacial nociceptive information directly to the thalamus and PBN via axon collaterals.

## Introduction

It is well established that the spinal trigeminal nucleus (Vsp), which contains the oral subnucleus (Vo), interpolar subnucleus (Vi) and caudal subnucleus (Vc), sends dense projection fibers not only to the thalamus [1, 2] but also the parabrachial nucleus (PBN) in the rat [3]. Previous studies have indicated that approximately 20% of neurons in the Vc had collateral projections to the contralateral thalamus and ipsilateral PBN, and more than 90% are distributed in lamina I (marginal zone) [4]. It is reported that the neurons in lamina I of the Vc respond exclusively or maximally to noxious orofacial stimuli [5].

Glutamate is the main excitatory neurotransmitter in the central nervous system. Since the successful cloning of the vesicular glutamate transporter (VGLUT) in the late 1990s [6–9], three types of VGLUTs have been identified. Two types, VGLUT1 and VGLUT2, have been considered definitive markers for glutamatergic neurons [6, 7, 10–14]. The different distribution patterns of the two types of VGLUTs in the central nervous system suggest that they may play different functional roles [11]. Previous reports have indicated the different distribution patterns of VGLUT1 and VGLUT2 mRNA in the principal sensory trigeminal nucleus (Vp) and Vsp of rats [15–17], in which single Vp neurons that express both VGLUT1 and VGLUT2 mRNA constituted approximately 64% of glutamatergic Vp neurons and the majority of glutamatergic T-T projection neurons in the Vp co-express VGLUT1 and VGLUT2 mRNA. A previous study [15] also showed that the glutamatergic T-T projection neurons in the Vsp mainly express VGLUT2, whereas trigemino-cerebellar projection neurons mainly express VGLUT1, which occurs most frequently in the Vi, less in the Vp, and least in the Vo. However, no single neurons that express VGLUT1 or VGLUT2 mRNA sending collateral projections to both the thalamus and PBN were reported in the Vsp, although many VGLUT1 or VGLUT2-positive axon terminals were identified in the PBN, as previously reported [14]; moreover, whether collateral projection neurons that express VGLUT1 or VGLUT2 mRNA are involved in nociceptive signal transmission has not been reported. Thus, in the present study, we primarily examined (1) which of the two main isoforms of VGLUTs may be expressed in the T-T and T-P collateral projection neurons in each of the subdivisions of the Vsp via FISH histochemistry combined with tract tracing after microinjection of a retrograde tracer (tetramethylrhodamine dextran amine, TMR) or wheat germ agglutinin-horseradish peroxidase (WGA-HRP) into the thalamic region and injection of Fluoro-Gold (FG) into the PBN region, respectively, and (2) whether the collateral projection neurons in the Vsp may be related to nociceptive signal transmission. The nociceptive neurons were confirmed by immunoreactivity for Fos, the protein product of the *c-fos* proto-oncogene, after subcutaneous injection of formalin into the upper lips [18, 19, 20]. In addition, in the Vc, the collateral projection neurons in synaptic contact with calcitonin gene related peptide (CGRP)-like immunoreactive (-LI) axon terminals were also considered nociceptive [21]. The synaptic relationship of collateral projection neuronal profiles (FG- and WGA-HRP-labeled) to axon terminals that exhibited CGRP-LI was also examined using electron microscopy.

## Methods

### Animals

A total of 51 adult male rats (Sprague-Dawley) that weighed between 280 and 320 g (China SH, Xi’an, People’s Republic of China) were used in the current study. Animal use and care was approved by the Animal Care and Use Committee at the Fourth Military Medical University. Of these rats, 30 rats were used for dual retrograde tract-tracing combined with FISH histochemistry or immunofluorescence histochemistry, 15 rats were used for anterograde tract-tracing combined with immunofluorescence histochemistry, and 6 rats were used for electron microscopy.

### Microinjection of TMR and FG solution into the thalamus and PBN for retrograde tract-tracing

Following an intraperitoneal injection of sodium pentobarbital (40 mg/kg body weight), the anaesthetized rats were placed in a stereotaxic frame (NARISHIGE, Japan). Using a glass micropipette (internal tip diameter: 15–25 μm) that was attached to a 1 μl Hamilton microsyringe, 0.6–0.8 μl of 10% TMR (D-3308, 3000 MW; Molecular Probes, Eugene, OR, USA) dissolved in 0.1 M of citrate-NaOH (pH 3.0) was injected into the right thalamus, and 0.2 μl of 4% FG (80,014, Biotium, Hayward, CA, USA) dissolved in normal saline was injected into the left PBN. After each injection, the glass micropipette was maintained in place for 15 min. All 30 rats injected with TMR and FG were allowed to survive for 7 days. Furthermore, the rats were equally divided into two groups. While lightly anaesthetized with ethyl ether, 0.1 ml of normal saline was injected into the upper lip ipsilateral to the FG injection site of the 15 rats in the first group, whereas the rats in the second group were subcutaneously injected with 0.1 ml of 4% formalin dissolved in normal saline into the upper lip ipsilateral to the FG injection site. The animals subsequently survived for 2 h prior to euthanasia. The results of the tract tracing were obtained from 6 rats in which the tracer was injected properly into the two target areas; the remaining 24 rats were discarded because of inappropriate injection sites.

### FISH histochemistry combined with FG and TMR retrograde tract tracing

The riboprobes for VGLUT1 mRNA and VGLUT2 mRNA have previously been described [22]. A cDNA fragment of VGLUT1 (nucleotides 855–1788; GenBank accession number XM_133432.2) or VGLUT2 (nucleotides 848–2044; GenBank accession number NM_080853.2) was cloned into a vector pBluescript II KS (+) (Stratagene, La Jolla, CA, USA). Using the linearized plasmids as templates, we subsequently synthesized the digoxigenin (DIG)-labeled antisense single-strand RNA probes with a DIG RNA labeling kit (Roche Diagnostic, Basel, Switzerland).

Seven days after the injection of FG and TMR, the 15 rats in the first group were re-anaesthetized intraperitoneally with an overdose of sodium pentobarbital (60 mg/kg); the rats were then transcardially perfused with 0.01 M of sodium phosphate-buffered 0.9% (*w*/*v*) saline (PBS, pH 7.3), followed by 500 ml of 4% (w/v) paraformaldehyde in 0.1 M of phosphate buffer (PB, pH 7.3). After perfusion, the brains were further postfixed in 4% paraformaldehyde for 3 days at 4 °C. Cryoprotected with 30% (w/v) sucrose in 0.1 M of PB for 2 days, the whole brains were serially cut into 20-μm-thick transverse sections using a freezing microtome (Leica CM1950; Leica, Germany).

The sections were divided into 7 series of alternate serial sections. One series of sections was directly mounted onto clean glass slides and air dried. In these sections, the location and extent of the TMR and FG injection sites, as well as the distribution of TMR- and FG-labeled neurons in the Vsp, were observed with an epifluorescence microscope (BX60; Olympus, Tokyo, Japan) under an appropriate filter for TMR (excitation 540–552 nm; emission 575–625 nm) and FG (excitation 360–370 nm; emission≥395 nm).

An additional three series of the sections were used for FISH combined with immunofluorescence histochemistry. In brief, free-floating sections were treated with 2% H_2_O_2_ in 0.1 M of PB for 10 min at room temperature (RT). After rinsing with 0.1 M of PB, the sections were incubated in 0.3% Triton-X100 in 0.1 M of PB at RT for 20 min and 10 min in acetylation solution, which consisted of 0.25% (*v*/v) acetic anhydride in 0.1 M of triethanolamine. After rinsing for 10 min twice, the sections were pre-hybridized for 1 h at 58 °C in a hybridization buffer, which contained 50% (*v*/v) formamide, 5 × saline sodium citrate (SSC; 1×SSC = 0.15 M of NaCl and 0.015 M of sodium citrate, pH 7.0), 2% (*w*/*v*) blocking reagent (Roche Diagnostics), 0.1% (w/v) N-lauroylsarcosine (NLS) and 0.1% (w/v) sodium dodecyl sulfate (SDS). VGLUT1 or VGLUT2 riboprobes were subsequently added into the hybridization system with a final concentration of 1 μg/ml and hybridized at 58 °C for 20 h. After two washes for 20 min at 55 °C with wash buffer, which contained 2 × SSC, 50% (v/v) formamide and 0.1% (*w*/*v*) NLS, the hybridized sections were incubated with 20 μg/ml ribonuclease A for 30 min at 37 °C in a mixture of 10 mM of Tris–HCl (pH 8.0), 1 mM of EDTA and 0.5 M of NaCl, followed by 2 washes for 20 min at 37 °C in 0.2 × SSC that contained 0.1% (*w*/*v*) NLS. The sections were subsequently incubated overnight at room temperature with a mixture of 0.5 μg/ml peroxidase-conjugated anti-digoxigenin sheep antibody (11–207–733-910; Roche Diagnostics, Basel, Switzerland), 1 μg/ml guinea pig anti-FG antibody (NM-101, Protos Biotech Corporation, NY, USA) and 1 μg/ml rabbit anti-TMR antibody (A-6397, Invitrogen, Eugene, OR, USA) in 0.1 M of Tris–HCl (pH 7.5)-buffered 0.9% (w/v) saline (TS 7.5) that contained 1% blocking reagent (TSB). To amplify the VGLUT1 or VGLUT2 mRNA hybridization signals, we performed the biotinylated tyramine (BT)-glucose oxidase (GO) amplification method with a reaction mixture that consisted of 1.25 μM of BT, 3 μg/ml GO, 2 mg/ml β-D-glucose, and 1% bovine serum albumin (BSA) in 0.1 M of PB for 30 min. The sections were subsequently treated with a mixture of 10 μg/ml Fluorescein Avidin D (A-2001; Vector, Burlingame, CA, USA), 10 μg/ml Alexa 647-conjugated goat anti-guinea pig IgG antibody (A-21450; Invitrogen) and 10 μg/ml Alexa 594-conjugated donkey anti-rabbit antibody (A-21207; Invitrogen) in TSB for 4 h.

The FISH was also performed using the sense probe (the third and fourth series of sections); however, no hybridization signals were detected in these sections.

### CGRP-immunoreactive axonal varicosities in the Vc were detected in apposition to FG- and TMR-labeled neuronal profiles by triple-immunofluorescence histochemistry

The fifth series of the sections through the Vc was incubated overnight with a mixture of 1 μg/ml guinea pig anti-FG antibody (Protos Biotech Corporation), 0.5 μg/ml rabbit anti-TMR antibody(Invitrogen), and 1 μg/ml goat anti-CGRP antibody (ab36001; Abcam) in PBS-XCD. After three washes with PBS, the sections were treated with 10 μg/ml biotinylated donkey anti-goat IgG (AP180B, Millipore, Temecula, CA, USA). The sections were then further incubated overnight with a mixture of 10 μg/ml Alexa 647-conjugated goat anti-guinea pig IgG antibody (Invitrogen), 10 μg/ml Alexa 594-conjugated donkey anti-rabbit IgG antibody (Invitrogen), and 10 μg/ml Fluorescein Avidin D (Vector) in PBS that contained 5% (*v*/v) normal donkey serum.

The sixth sections, which contained the Vsp of the rats injected with FG and TMR, were used to conduct the control experiments for immunofluorescence histochemistry, in which CGRP antibody was omitted. Under these conditions, no immunoreactivity for the omitted antibody was observed.

### Detection of Fos immunoreactivity in Vc neurons labeled with FG and TMR by triple-immunofluorescence histochemistry

The last sections through the Vc of the rats in the first group and the sections through the Vc from the second group injected with FG, TMR, and formalin were also processed for triple-immunofluorescence histochemistry for Fos, TMR and FG. Briefly, the sections were incubated with (1) a mixture of 1 μg/ml mouse anti-Fos antibody (ab 208,942, Abcam, Cambridge, MA, USA), 1 μg/ml guinea pig anti-FG antibody (NM-101, Protos Biotech Corporation) and 0.5 μg/ml rabbit anti-TMR antibody (A-6397, Invitrogen) in PBS that contained 0.3% (*v*/v) Triton X-100, 0.25% (*w*/*v*) λ-carrageenan, and 3% (v/v) donkey serum (PBS-XCD) overnight at room temperature; (2) 10 μg/ml biotinylated donkey anti-mouse IgG (AP192B, Millipore, Temecula, CA, USA); and (3) a mixture of 10 μg/ml Alexa 647-conjugated goat anti-guinea pig IgG antibody (A-21450, Invitrogen), 10 μg/ml Alexa594-conjugated donkey anti-rabbit IgG antibody (A-21207, Invitrogen), and 10 μg/ml Fluorescein Avidin D (A-2001; Vector) in PBS that contained 3% (v/v) normal donkey serum.

Another series of sections from the second group was used to conduct the control experiments for immunofluorescence histochemistry, in which the Fos antibody was omitted. Under these conditions, no immunoreactivity for the omitted antibody was observed.

### Immunofluorescence histochemistry combined with biotinylated dextran amine (BDA) anterograde tract tracing

Anterograde tract tracing from the Vsp to the PBN regions was performed using BDA (D1956, Invitrogen) in 15 rats. In each rat, 0.2 μl of 2% (*w*/*v*) BDA in distilled water was injected into the Vo (5 rats), Vi (5 rats) or Vc (5 rats). After a period of 5 days, the rats injected with BDA were sacrificed. Similar to the procedure of the FG and TMR injection experiments, the brainstem of the rats was cut into transverse sections in series, and one series of sections through Vo, Vi or Vc was directly incubated with Fluorescein Avidin D to detect the location and extent of the BDA injection site.

The sections through the PBN from the rats that had a proper BDA injection site were subsequently incubated overnight at room temperature with a mixture of 1 μg/ml mouse anti-NeuN antibody (MAB377; Millipore, Billerica, MA) and 2 μg/ml guinea pig anti-VGLUT2 (135,404, Synaptic Systems, Goettingen, German) in PBS that contained PBS-XCD, followed by 6 h at RT with a mixture of 5 μg/ml Fluorescein Avidin D (Vector), 5 μg/ml Alexa 594-conjugated goat anti-guinea pig IgG antibody (A-11076, Invitrogen) and 5 μg/ml Alexa 647-conjugated donkey anti-mouse IgG antibody (A-31571, Invitrogen).

### Confocal laser scanning microscopy for the immunofluorescence stained sections

After incubation, all immunofluorescence stained sections were observed under a confocal laser scanning microscope (FV1000; Olympus, Tokyo, Japan) with appropriate laser beams and filter sets for fluorescein (excitation 488 nm, emission 510–530 nm), TMR and Alexa 594 (excitation 543 nm, emission 590–615 nm) or Alexa 647 (excitation 633 nm, emission 650 nm). We captured the digital images with an FV10-ASW 1.6 from Olympus; following modifications (15–20% contrast enhancement) in Photoshop CS4 (Adobe Systems, San Jose, CA), these images were saved as TIFF files.

### Cell counting and statistics

In the 3 rats in which the FG injection into the PBN and TMR injection into the thalamus were successful, the TMR- and FG-labeled cell bodies of neurons that expressed VGLUT1 or VGLUT2 mRNA were identified by FISH combined with retrograde tract tracing. For these counts, in each rat, we selected 15 sections that covered the whole rostral-caudal axis of the Vsp, 5 sections of the Vo, 5 sections of the Vi and 5 sections of the Vc. In the other 3 rats, after the TMR and FG were administered and the formalin was subcutaneously injected into the upper lip ipsilateral to the FG injection site, the TMR- or FG-labeled cell bodies of neurons that expressed Fos immunoreactivity were identified by immunohistochemistry combined with retrograde tract tracing. For these counts, in each rat, 10 sections that covered the whole rostral-caudal axis of the Vc were selected. The target areas (Vo, Vi or Vc) were subsequently photographed with a confocal laser microscope under a 10× objective, and the number of neurons double-labeled with TMR/FG, TMR/VGLUT1 mRNA, TMR/VGLUT2 mRNA, FG/VGLUT1 mRNA, or FG/VGLUT2 mRNA, and the neurons triple-labeled with TMR/FG/VGLUT1, TMR/FG/VGLUT2 or TMR/FG/Fos were counted based on these photographs. In all experiments, only cells with a clear nucleus were counted.

For statistics, the numbers of the counted cells of each rat were initially summed and then averaged among the three rats. All data are presented as the mean ± standard deviation (SD). *Student’s t* test was used to determine whether the number of Fos immuno-reactive cells in the Vc of the rats after formalin injection was significantly different from that in the rats injected with normal saline. For cell counting in the Vc, the identification of each lamina of the Vc referred to the rat brain atlas by Paxinos [23] and the book “The Rat Nervous System” by Paxinos [24]. The layers consist of a marginal layer (lamina I) and substantia gelatinosa (lamina II), which together comprise the superficial laminae, and a deeper magnocellular layer (laminae III and IV; a separate lamina III is not obvious in the rat; however, it is typically included in the magnocellular layer).

### Triple immune-electron microscopy showed CGRP-immunoreactive terminals in synaptic contact with WGA-HRP- and FG-labeled neuronal profiles in Vc

Six rats were injected with WGA-HRP and FG for electron microscopy. In each rat, 0.2 μl of FG was injected by pressure into the left PBN as previously described. After 4 days, the rats were re-anaesthetized with sodium pentobarbital (40 mg/kg body weight) and stereotaxically injected with 0.6 μl of 1% WGA-HRP (PL-1026, Vector) into the right thalamus of the FG injected rats. The procedures for the stereotaxic microinjection of the WGA-HRP solution were the same as described for FG. After the injection of WGA-HRP, the rats were allowed to survive for 3 days.

The rats were deeply anaesthetized and transcardially perfused with 200 ml of 4% (*w*/*v*) paraformaldehyde, 0.1% (w/v) glutaraldehyde, and 15% (*v*/v) saturated picric acid in 0.1 M of PB. The brainstems were serially cut into 50-μm-thick transverse sections with a Vibratome (Microslicer DTM-1000; Dosaka EM, Kyoto, Japan). The staining of WGA-HRP was processed using tetramethylbenzidine (TMB) with sodium tungstate as a stabilizer [25], and the WGA-HRP reaction products were further intensified with DAB/cobalt/H_2_O_2_ solution. The sections that contained the injection sites were subsequently mounted onto glass slides for the conformation of the injection site. The sections through the Vc from 3 animals with both WGA-HRP and FG injection sites restrained in the target area were selected and incubated in a mixture of 25% (*w*/*v*) sucrose and 10% (*v*/v) glycerol in 0.05 M of PB for 1 h. The sections were transiently frozen and thawed with liquid nitrogen. Following incubation at room temperature with 0.05 M of Tris-HCl-buffered saline (TBS; pH 7.4) that contained 20% (v/v) normal donkey serum for 1 h, the sections were processed for double immunolabeling of FG and CGRP. In brief, the sections were incubated at room temperature overnight with 1 μg/ml rabbit anti-FG antibody (A153-I, Millipore) and 1 μg/ml mouse anti-CGRP antibody (ab81887, Abcam) in TBS that contained 2% (v/v) normal donkey serum (TBS-D). After rinsing with TBS, the sections were incubated in TBS-D with 10 μg/ml 1.4-nm gold-particle-conjugated goat anti-rabbit IgG antibody (2004; Nanoprobes, Stony Brook, NY, USA) and 10 μg/ml biotin-conjugated donkey anti-mouse IgG antibody (Millipore) overnight. The sections were subsequently treated with 1% (*w*/*v*) glutaraldehyde in 0.1 M of PB (pH 7.4) for 10 min and rinsed with distilled water. An HQ Silver Kit (2012; Nanoprobes) was subsequently employed to perform silver enhancement. The sections were then incubated with a 1:50-diluted Elite ABC Kit (PK-2101, Vector) in 0.05 M of TBS for 6 h and further treated with 0.02% (w/v) 3,3-diaminobenzidine tetrahydrochloride (DAB; D5637, Sigma, St. Louis, MO, USA) and 0.3% (*v*/v) H_2_O_2_ in 0.05 M of Tris–HCl (pH 7.6) for 30 min. The sections were subsequently incubated in 1% (w/v) OsO4 in 0.1 M of PB (pH 7.4) for 35 min and counterstained in 70% ethanol that contained 1% (w/v) uranyl acetate for 1 h. With dehydration, the sections were mounted onto silicon-coated glass slides, and 1 was embedded in epoxy resin (Durcupan; Fluka, Buchs, Switzerland). Once the resin polymerized, section fragments that contained the superficial layer of the Vc were removed from the resin. The selected tissue fragments were further cut into 60-nm-thick sections using an ultramicrotome (Reichert-Nissei Ultracut S; Leica). The ultrathin sections were then mounted onto single-slot grids coated with pioloform membranes and detected with a JEM-1400 electron microscope (JEM, Tokyo, Japan).

## Results

**Fig. 1:**
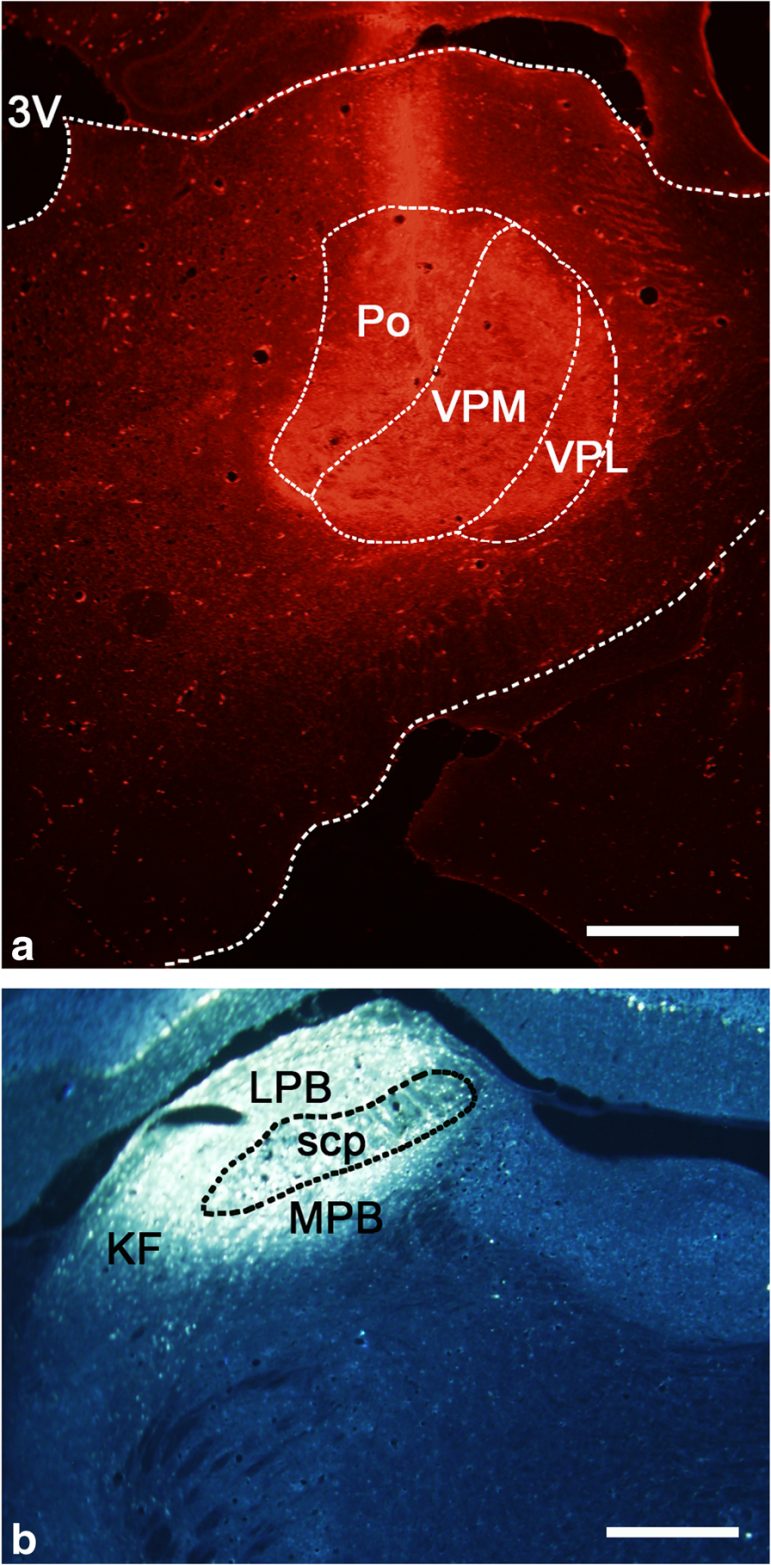
Photomicrographs of a section through the middle level of a TMR injection site in the right side of the thalamus, involving the VPM and Po (**a**), and an FG injection site in the left side of the PBN (**b**) in R7. *Scale bar* 500 μm (**a**); 300 μm (**b**)

**Fig. 2:**
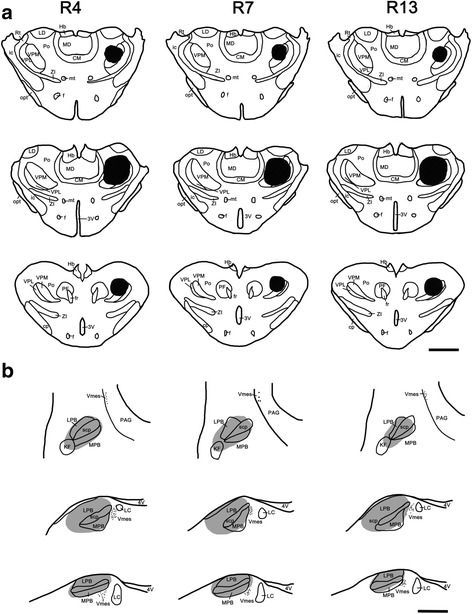
Projection drawings indicate the sites of TMR injection into the thalamus and FG injection into the PBN in three rats (R4, R7, and R13). **a** Three different sections from rostral to caudal of the thalamus show the TMR injection sites confined in the VPM, the Po and their vicinities. **b** FG was injected into the left PBN, with the densest core (blackened areas) covering parts throughout the PBN and the KF. *Scale bar* 2 mm (**a**); 1 mm (**b**)

Both a TMR injection into the thalamus and an FG injection into the PBN were performed in 30 rats. In the sites of the TMR or FG injections into the thalamus or PBN, a dense core of the tracer was surrounded by a diffuse halo of the same. The brightest injection areas were considered to represent the injection site. When a TMR injection site was confined within the targeted area in the VPM and Po and their immediate vicinities, the TMR injection into the thalamus was considered successful. When the lateral and medial parabrachial nuclei (LPB and MPB), including the Kölliker-Fuse nucleus, were involved in an injection site, the FG injection into the PBN region was considered successful. Thus, 6 of the 30 rats were considered to be successfully injected, as the TMR and FG injection sites both involved the target area (Figs. 1, 2 and 6). Although the location and extent of the injection sites in these 3 rats (R4, R7, and R13 in TMR and FG; R18, R25, and R29 in TMR, FG, Fos) were variable, the patterns of retrograde labeling in the Vsp were relatively similar.

### Dual retrograde tract tracing combined with FISH histochemistry for VGLUT1 or VGLUT2 mRNA

To examine whether Vsp neurons that express VGLUT1 or VGLUT2 mRNA may send their axon collaterals to the contralateral thalamus and ipsilateral PBN, retrograde tract tracing combined with FISH histochemistry for VGLUT1 or VGLUT2 mRNA was performed after TMR injection into the right thalamus and FG injection into the left PBN in R4, R7 and R13 (Figs. 1 and 2). Using confocal laser scanning microscopic detection, many TMR- or FG-labeled neurons were observed in the left Vsp (ipsilateral to the FG injection into the PBN; contralateral to the TMR injection into the thalamus), whereas only a small number were observed on the right side. Thus, all observations were focused on the left Vsp.

**Fig. 3:**
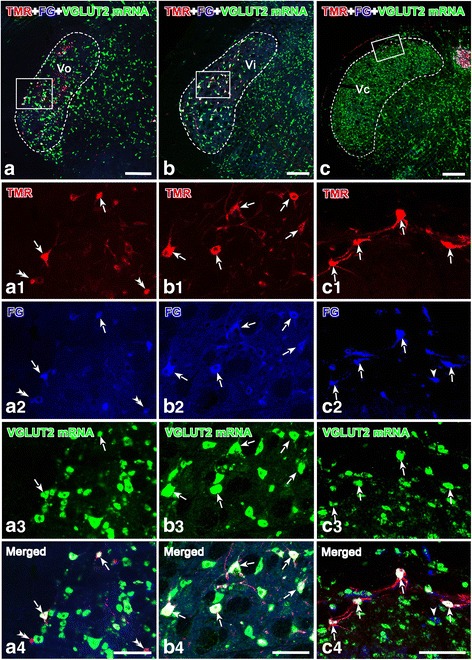
Collateral projection neurons to both the thalamus and the PBN labeled with hybridization signals for VGLUT2 mRNA in the Vo (**a**, a1-a4), Vi (**b**, b1-b4) and Vc (**c**, c1-c4). Some neurons are retrogradely labeled with TMR and FG. Immunoreactivity for TMR is visualized with Alexa 594 (*red*), and immunoreactivity for FG is visualized with Alexa 647 (*blue*), whereas the VGLUT2 mRNA hybridization signals are shown with fluorescein (*green*). Images (a1-a4, b1-b4 And c1-c4**)** are the magnified images of the framed areas in (**a**, **b** and **c**), respectively. The *arrows* in (a1-c4) indicate the cell bodies triply labeled with TMR/FG/VGLUT2 mRNA signals (*white*); the *double arrowheads* in a1-c4 indicate the cell bodies dually labeled with TMR and FG (*purple red*); the *single arrowheads* in (a1-c4) point to cell bodies singly labeled with FG (*blue*). *Scale bar* 300 μm (a-c); 100 μm (a1-c4)

**Fig. 4:**
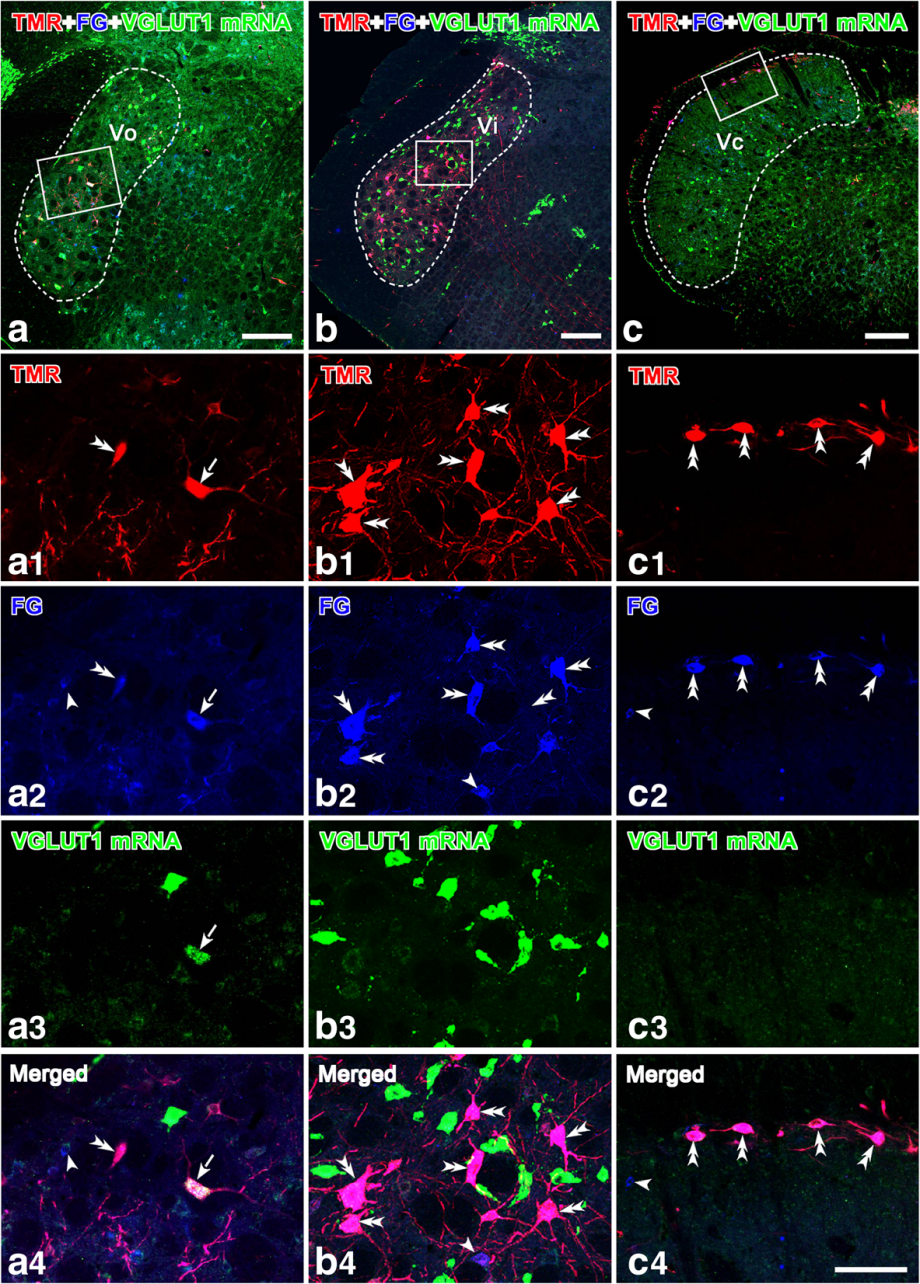
Collateral projection neurons to both the thalamus and the PBN labeled with hybridization signals for VGLUT1 mRNA in the Vo (**a**, a1-a4), Vi (**b**, b1-b4) and Vc (**c**, c1-c4). Some neurons are retrogradely labeled with TMR and FG. Immunoreactivity for TMR is visualized with Alexa 594 (*red*), and immunoreactivity for FG is visualized with Alexa 647 (*blue*), whereas the VGLUT1 mRNA hybridization signals are shown with fluorescein (*green*). Images (a1-a4), (b1-b4) and (c1-c4) are the magnified images of the framed areas in (**a**), (**b**) and (**c**), respectively. The *arrows* in (a1-a4) indicate the cell bodies triply labeled with TMR/FG/VGLUT1 mRNA signals (*white*); the *double arrowheads* in (a1-c4) indicate the cell bodies dually labeled with TMR and FG (*purple red*); the *single arrowheads* in (a1-c4) point to cell bodies singly labeled with FG (*blue*). *Scale bar* 300 μm (**a**-**c**); 100 μm (a1-c4)

In general, the results indicated that relatively more TMR-labeled neurons were observed in the Vi (Figs. 3b and 4b), and fewer neurons were scattered within the Vo (Figs. 3a and 4a) and Vc (Figs. 3c and 4c). FG-labeled neurons were distributed in the Vo with slight dominance at the caudal levels (Figs. 3a and 4a), whereas the ventral and dorsal parts at the rostro-caudal axis of the Vi showed slight dominance at the rostral levels (Figs. 3b and 4b). In the Vc, most of the TMR-labeled neurons were located in the superficial layer (Figs. 3c and 4c). Similar to the TMR-labeled neurons, the FG-labeled neurons in the Vc were mainly distributed in the superficial layer (Fig. 3c, c2, c4; Fig. 4c, c2, c4). There was a substantial number of neuronal cells with VGLUT2 mRNA signals distributed throughout the Vsp (Fig. 3c). In contrast, neurons with VGLUT1 mRNA signals were also abundant in the Vi (Fig. 4b, b3, b4), whereas only a small number were scattered in the Vo (Fig. 4a, a3, a4) and Vc (Fig. 4c, c3, c4). Moreover, the VGLUT1 mRNA-positive neurons were mainly distributed in the deep layer of the Vc (compare Fig. 4c with Fig. 3c).

Furthermore, cell counts were performed in the Vsp of the three rats with images obtained via confocal laser scanning microscopy (Tables 1 and 2). TMR/FG double-labeled neurons in the Vo, Vi and Vc accounted for (90.5 ± 1.8) %, (92.5 ± 1.9) % and (49.6 ± 1.0) % of the TMR-labeled cells, respectively, and (53.5 ± 2.5) %, (73.6 ± 1.2) % and (43.7 ± 1.9) % of the FG-labeled cells, respectively (Table 1). Overall, (88.2 ± 2.5) %, (92.0 ± 5.3) % and (64.5 ± 1.0) % of the TMR retrograde-labeled neurons and (6.0 ± 0.2) %, (22.2 ± 1.6) % and (0.7 ± 0.1) % of the VGLUT2 mRNA positive cells exhibited double labeling of TMR and VGLUT2 mRNA in the Vo, Vi and Vc, respectively (Table 1). Furthermore, (83.9 ± 1.6) %, (96.6 ± 1.1) % and (90.7 ± 2.9) % of the FG retrogradely labeled neurons and (9.7 ± 0.2) %, (29.2 ± 0.7) % and (1.1 ± 0.1) % of the VGLUT2 mRNA-positive cells exhibited double labeling for both FG and VGLUT2 mRNA (Table 1). TMR/FG/VGLUT2 triple-labeled neurons accounted for (81.7 ± 3.5) %, (86.0 ± 0.7) %, and (42.4 ± 0.9) % of the TMR-labeled neurons, (48.3 ± 1.7) %, (68.4 ± 0.3) % and (37.3 ± 1.6) % of the FG-labeled neurons, (5.6 ± 0.1) %, (20.7 ± 0.2) % and (0.4 ± 0.0) % of the VGLUT2 mRNA positive cells, and (90.3 ± 4.1) %, (93.0 ± 1.2) % and (85.4 ± 0.7) % of the TMR/FG double labeled neurons in the Vo, Vi and Vc (Table 1).

**Table 1:** Number of TMR, FG and VGLUT2 mRNA-labeled neurons in the Vo, Vi, and Vc of R4, R7 and R13

	Vo	Vi	Vc
(1)TMR	92 ± 8	176 ± 9	65 ± 8
(2)FG	155 ± 9	222 ± 11	73 ± 4.5
(3)VGLUT2 mRNA	1347 ± 96	732 ± 27	6285 ± 132
(4)TMR + FG	83 ± 9	163 ± 6	32 ± 3.3
(5)[(4)/(1)] × 100	(90.5 ± 1.8)%	(92.5 ± 1.9)%	(49.6 ± 1.0)%
(6)[(4)/(2)] × 100	(53.5 ± 2.5)%	(73.6 ± 1.2)%	(43.7 ± 1.9)%
(7)TMR + VGLUT2 mRNA	81 ± 6	162 ± 18	42 ± 5
(8)[(7)/(1)] × 100	(88.2 ± 2.5)%	(92.0 ± 5.3)%	(64.5 ± 1.0)%
(9)[(7)/(3)] × 100	(6.0 ± 0.2)%	(22.2 ± 1.6)%	(0.7 ± 0.1)%
(10)FG + VGLUT2 mRNA	130 ± 10	214 ± 13	66 ± 6
(11)[(10)/(2)] × 100	(83.9 ± 1.6)%	(96.6 ± 1.1)%	(90.7 ± 2.9)%
(12)[(10)/(3)] × 100	(9.7 ± 0.2)%	(29.2 ± 0.7)%	(1.1 ± 0.1)%
(13)FG + TMR + VGLUT2 mRNA	75 ± 6	152 ± 7	27 ± 3
(14)[(13)/(1)] × 100	(81.7 ± 3.5)%	(86.0 ± 0.7)%	(42.4 ± 0.9)%
(15)[(13)/(2)] × 100	(48.3 ± 1.7)%	(68.4 ± 0.3)%	(37.3 ± 1.6)%
(16)[(13)/(3)] × 100	(5.6 ± 0.1)%	(20.7 ± 0.2)%	(0. 4 ± 0.0)%
(17)[(13)/(4)] × 100	(90.3 ± 4.1)%	(93.0 ± 1.2)%	(85.4 ± 0.7)%

TMR is injected in the right thalamus, and FG is injected into the left PBN. The cell body counts were performed for three rats on the left side. Each figure showing the number of cell bodies of one animal was the average of the three rats (5 sections from each rat). The sections were 20 μm thick. FG, TMR and VGLUT2 mRNA represent all FG-, TMR- and VGLUT2 mRNA-labeled neurons; data are shown as the mean ± standard deviation (SD)

**Table 2:** Number of TMR, FG and VGLUT1 mRNA-labeled neurons in the Vo, Vi, and Vc of R4, R7 and R13

	Vo	Vi	Vc
(1)TMR	90 ± 8	180 ± 10	63 ± 7
(2)FG	155 ± 9	223 ± 8	71 ± 5.3
(3)VGLUT1 mRNA	136 ± 9	634 ± 15	241 ± 12
(4)TMR + FG	81 ± 8	172 ± 12	31 ± 4.5
(5)[(4)/(1)] × 100	(89.5 ± 1.5)%	(95.1 ± 1.3)%	(49.0 ± 2.6)%
(6)[(4)/(2)] × 100	(51.8 ± 2.4)%	(77.2 ± 2.8)%	(43.2 ± 3.4)%
(7)TMR + VGLUT1 mRNA	17 ± 4	1 ± 0.8	3 ± 2
(8)[(7)/(1)] × 100	(19.1 ± 2.5)%	(0.5 ± 0.4)%	(4.5 ± 2.1)%
(9)[(7)/(3)] × 100	(12.6 ± 1.9)%	(0.2 ± 0.1)%	(1.2 ± 0.6)%
(10)FG + VGLUT1 mRNA	22 ± 4	3 ± 1	3 ± 2
(11)[10)/(2)] × 100	(14.1 ± 2.4)%	(1.3 ± 0.7)%	(4.1 ± 2.0)%
(12)[(10)/(3)] × 100	(16.1 ± 2.6)%	(0.5 ± 0.2)%	(1.2 ± 0.6)%
(13)TMR + FG + VGLUT1 mRNA	24 ± 5	0	1 ± 0
(14)[(13)/(1)] × 100	(26.8 ± 3.0)%	0	(1.0 ± 0.7)%
(15)[(13)/(2)] × 100	(15.5 ± 2.2)%	0	(0.9 ± 0.6)%
(16)[(13)/(3)] × 100	(17.7 ± 2.4)%	0	(0.3 ± 0.2)%
(17)[(13)/(4)] × 100	(29.9 ± 3.0)%	0%	(2.0 ± 1.4)%

TMR is injected into the right thalamus, and FG is injected into the left PBN. The cell body counts were performed for three rats on the left side. Each figure showing the number of cell bodies of one animal was the average of the three rats (5 sections from each rat). The sections were 20 μm thick. FG, TMR and VGLUT1 mRNA represent all FG-, TMR- and VGLUT1 mRNA-labeled neurons; data are shown as the mean ± standard deviation (SD)

Moreover, (19.1 ± 2.5) %, (0.5 ± 0.4) % and (4.5 ± 2.1) % of the TMR retrogradely labeled neurons and (12.6 ± 1.9) %, (0.2 ± 0.1) % and (1.2 ± 0.6) % of the VGLUT1 mRNA positive cells exhibited dual labeling of TMR/VGLUT1 mRNA in the Vo, Vi and Vc, respectively (Table 2). Overall, (14.1 ± 2.4) %, (1.3 ± 0.7) % and (4.1 ± 2.0) % of the FG retrogradely labeled neurons and (16.1 ± 2.6) %, (0.5 ± 0.2) % and (1.2 ± 0.6) % of the VGLUT1 mRNA-positive cells were double labeled for both FG and VGLUT1 mRNA (Table 2). TMR/FG/VGLUT1 triple-labeled neurons comprised (26.8 ± 3.0) % and (1.0 ± 0.7) % of the MR-labeled neurons, (15.5 ± 2.2) % and (0.9 ± 0.6) % of the FG-labeled neurons, (17.7 ± 2.4) % and (0.3 ± 0.2) % of the VGLUT1 mRNA positive cells, and (29.9 ± 3.0) % and (2.0 ± 1.4) % of the FG/TMR double-labeled neurons in the Vo and Vc, respectively (Table 2). In the Vi, no TMR/FG/VGLUT1 triple-labeled neurons were identified; however, some neurons showed TMR/FG, TMR/VGLUT1 or FG/VGLUT1 double-labeling (Table 2).

Thus, these data indicated that the vast majority of the Vo, Vi and Vc neurons that projected both ipsilaterally to the PBN and contralaterally to the thalamus by way of axon collaterals were VGLUT2 mRNA-positive (Table 1), whereas a limited number expressed VGLUT1 mRNA (Table 2).

### Immunofluorescence histochemistry combined with anterograde tract-tracing after BDA injection into the Vo, vi and Vc

**Fig. 5:**
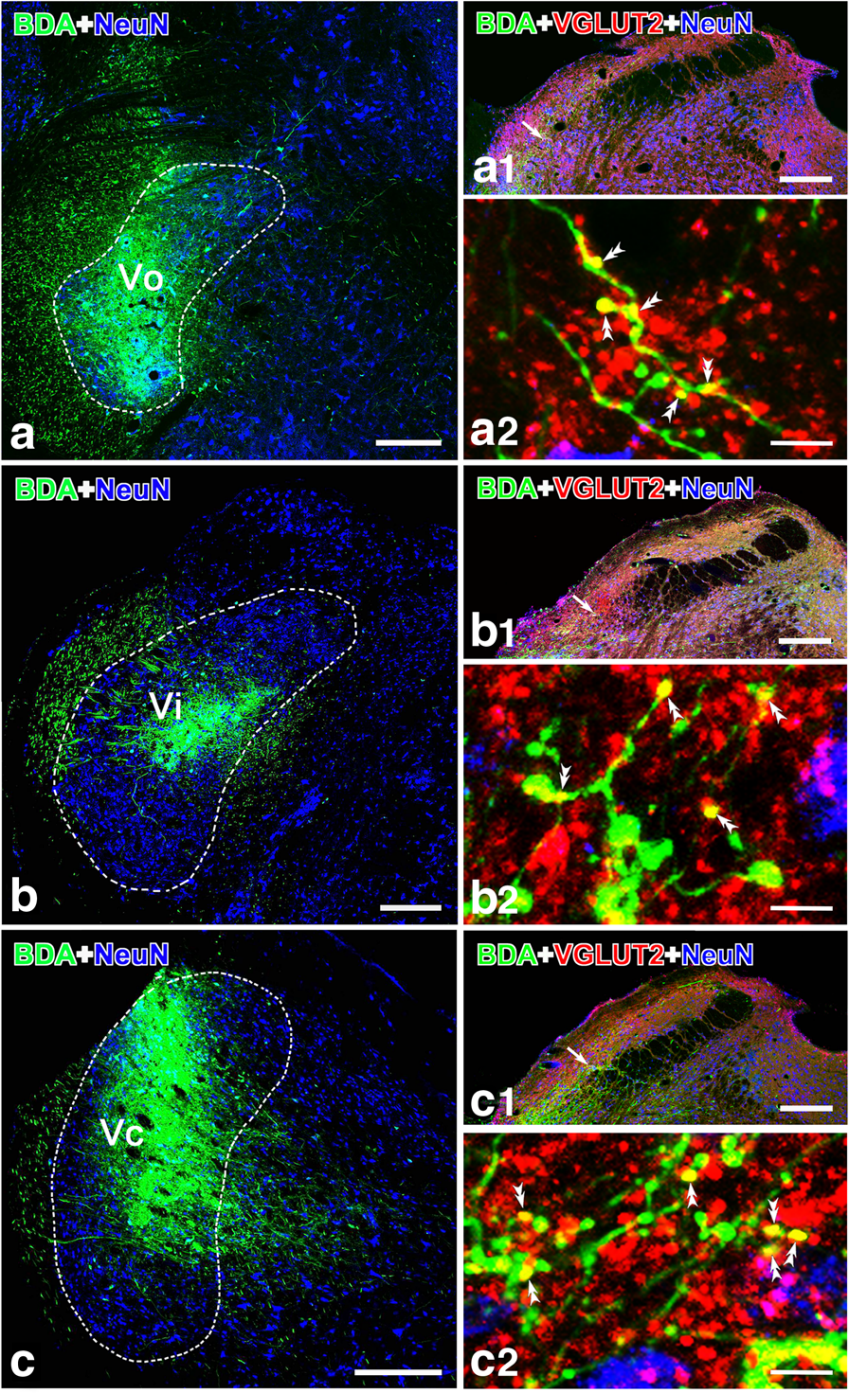
After the BDA injection into the Vo (**a**), Vi (**b**) or Vc (**c**), many BDA-labeled terminals in the ipsilateral PBN showed VGLUT2-LI (Vo: a1-a2; Vi: b1-b2; Vc: c1-c2). The site of BDA injection and the BDA anterogradely labeled terminals are visualized with fluorescein (*green*). VGLUT2-LI axon terminals are visualized with Alexa 594 (*red*), and neuronal cell bodies showing NeuN-LI are visualized with Alexa 647 (*blue*). The areas indicated by the short arrow in (a1), (b1) and (c1) are magnified in (a2), (b2) and (c2), respectively. The *double arrowheads* in (a2), (b2) and (c2) indicate axon terminals dually labeled with BDA and VGLUT2-LI (*yellow*). *Scale bar* 300 μm (**a**, **b** and **c**); 200 μm (a1, b1 and c1); 5 μm (a2, b2 and c2)

Anterograde tract-tracing experiments combined with immunofluorescence histochemistry for VGLUT2-LI were performed in 15 rats after unilateral injection of BDA into the Vo, Vi and Vc (Fig. 5a-c); the BDA injection sites were also immunostained with NeuN. The site of the BDA injection in the rats covered nearly the complete extent of the Vo (Fig. 5a), Vi (Fig. 5b) and Vc (Fig. 5c). In these experiments, the distribution of BDA-labeled axons and terminals in the PBN was very similar (Fig. 5a1, b1, c1). Anterograde labeling was present bilaterally in the PBN and KF, with a clear ipsilateral predominance. The highest density of BDA-labeled axon terminals was present caudally in the KF and in the ventral portion of the LPB (Fig. 5a1, b1, c1). The sections stained by triple-immunofluorescence labeling for VGLUT2/NeuN/BDA (for the rats injected with BDA to the Vo, Vi and Vc) were subsequently examined under a confocal laser scanning microscope, and the results showed that many BDA-labeled small granules in the PBN and KF showed VGLUT2 immunoreactivity (Fig. 5a2, b2, c2).

### Immunoreactivities for Fos in the Vc after subcutaneous injection of formalin into the rat upper lip

**Fig. 6:**
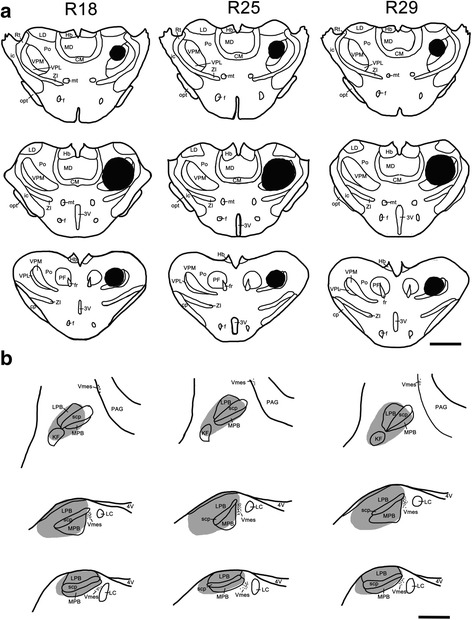
Projection drawings of the sites of TMR injection into the thalamus and FG injection into the PBN in three rats (R18, R25, and R29) that received formalin injection prior to perfusion. **a** Three different sections from rostral to caudal of the thalamus show the TMR injection into the right side of the thalamus site involving the VPM, the Po and their vicinities. **b** FG was injected into the left PBN, with the densest core (blackened areas) covering parts throughout the PBN and the KF. *Scale bar* 2 mm (**a**); 1 mm (**b**)

**Fig. 7:**
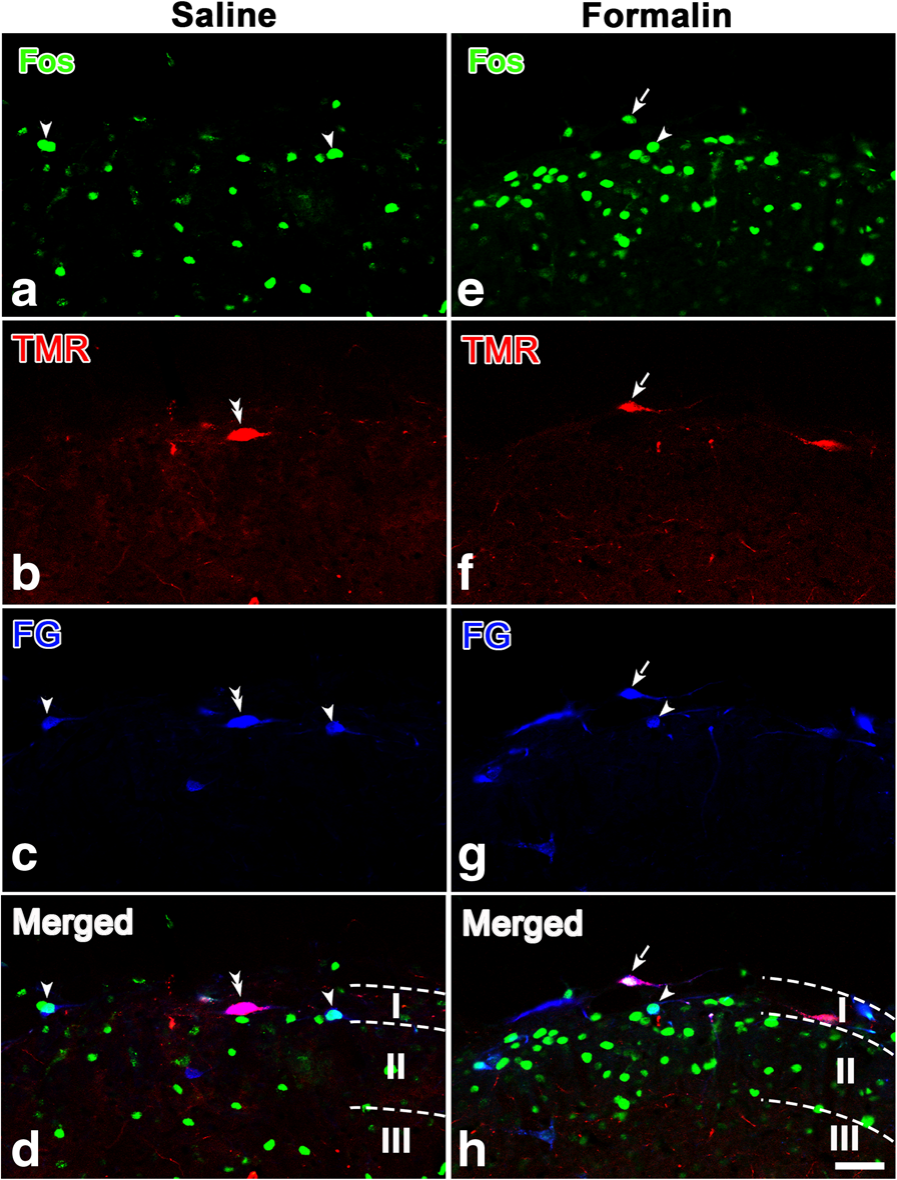
Vc neurons that send axon collaterals to both the thalamus and PBN are Fos positive after a subcutaneous 4% formalin injection into the left upper lip. TMR was injected into the right VPM and Po of the thalamus, and FG was injected into the left PBN. TMR, FG and Fos immunoreactivity in the Vc are visualized with Alexa 594 (*red*), Alexa 647 (*blue*) and fluorescein (*green*), respectively. **a-d** Representative section from animals injected with normal saline (R4, R7, and R13, Fig. 2) and showing triple-labeled with TMR, FG and Fos. **e-h** Representative section from animals injected formalin (R18, R25, and R29, Fig. 6) and showing triple-labeled with TMR, FG and Fos. The *double arrowheads* in (**b**, **c** and **d**) indicate neurons double-labeled with TMR/FG. The *single arrowheads* in (**a**-**h**) indicate neurons double-labeled with FG and Fos. The arrows in e-h indicate neurons triple-labeled with TMR/FG/Fos. I, II and III in (**d**) and (**h**) represent the laminae I, II and III of Vc. *Scale bar* 50 μm

**Fig. 8:**
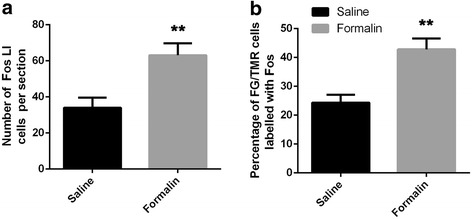
Comparison of the number of Fos-like immunoreactive (LI) neuronal cells in the superficial layer (laminae I and II) of the Vc in rats injected with normal saline and rats injected with formalin. **a** Compared with rats injected with normal saline, the number of Fos-LI cells per section in the superficial layer of the Vc significantly increased after the injection of formalin into the upper lip of rats. **b** The percentage of FG/TMR double-labeled neurons in one section in the superficial layer of the Vc showed Fos-LI substantially increased in rats injected with formalin. **P < 0.001, 30 sections from 3 rats

To examine whether the Vc collateral projection neurons previously identified may be involved in the transmission of orofacial nociceptive information, retrograde tract tracing combined with Fos  immunohistochemistry was performed after TMR injection into the right thalamus and FG injection into the left PBN. Similar to the first group that was injected with normal saline in the upper lip, 3 rats in the second group were also considered successful, as both of the injection sites of FG and TMR were restricted in the PBN and thalamus, respectively (R18, R25, and R29, Fig. 6). The results showed that Fos-LI cell bodies in the superficial layer (laminar I and laminar II) of the left Vc in the rats injected with formalin (Fig. 7e-h) significantly increased (Fig. 8a, *P*< 0.001, 30 sections from 3 rats) compared with those in the rats injected with normal saline (Fig. 7a-d). Moreover, the percentage of FG/TMR dual-labeled neurons in the superficial layer that showed Fos LI also significantly increased [(24.3 ± 2.8) % to (42.8 ± 3.8) %, *P* < 0.001, 30 sections from 3 rats] with the formalin injection (Fig. 8b). In detail, for the animals injected with formalin, (29.3 ± 0.4) % and (24.5 ± 1.7) % of the Fos  labeled Vc neurons were identified in laminae I and II, respectively, and (46.2 ± 0.5) % was distributed in lamina III (Table 3). TMR/FG double-labeled neurons were mainly distributed in lamina I of the Vc, with (64.0 ± 4.4) % of the total double-labeled neurons in lamina I and only (15.5 ± 6.5) % and (22.1 ± 2.9) % scattered in laminae II and III, respectively. In addition, TMR/FG/Fos triple-labeled neurons were mainly distributed in lamina I of the Vc and accounted for (77.8 ± 2.1) % of the total triple-labeled neurons in the Vc (Table 3).

**Table 3:** Number of neurons labeled with FG, TMR and (or) Fos in the Vc of R18, R25 and R29

	Lamina I	Lamina II	Lamina III	Total
(1)TMR	92 ± 9 (59.3 ± 1.5)%	12 ± 3 (7.9 ± 2.5)%	51 ± 7 (33.1 ± 0.3)%	142 ± 18
(2)FG	81 ± 8 (57.5 ± 1.8)%	21 ± 4 (15.5 ± 4.0)%	39 ± 6 (27.6 ± 0.7)%	155 ± 18
(3)Fos	344 ± 18 (29.3 ± 0.4)%	287 ± 11 (24.5 ± 1.7)%	541 ± 16 (46.2 ± 0.5)%	1172 ± 46
(4)TMR + FG	29 ± 6 (64.0 ± 4.4)%	7 ± 2 (15.5 ± 6.5)%	11 ± 4 (22.1 ± 2.9)%	47 ± 12
(5)TMR + FG + Fos	13 ± 4 (77.8 ± 2.1)%	2 ± 1 (14.2 ± 7.0)%	2 ± 0 (10.4 ± 1.6)%	16 ± 5
(6)[(5)/(4)] × 100	(42.1 ± 5.8)%	(29.4 ± 3.4)%	(16.4 ± 3.1)%	(34.4 ± 2.4)%

TMR is injected into the collateral thalamus, and FG is injected into the ipsilateral PBN. The cell body counts were performed for three rats on the ipsilateral side of the FG injection site. Each figure showing the number of cell bodies of one animal was the average of the three rats (10 sections from each rat). The sections were 20 μm thick. FG, TMR and Fos represent all FG-, TMR- and Fos-labeled neurons; data are shown as the mean ± standard deviation (SD)

### CGRP-like immunopositive axon terminals formed close contacts or asymmetric synapses with the neuronal soma or dendrites of the collateral projections into the thalamus and LPB in Vc

**Fig. 9:**
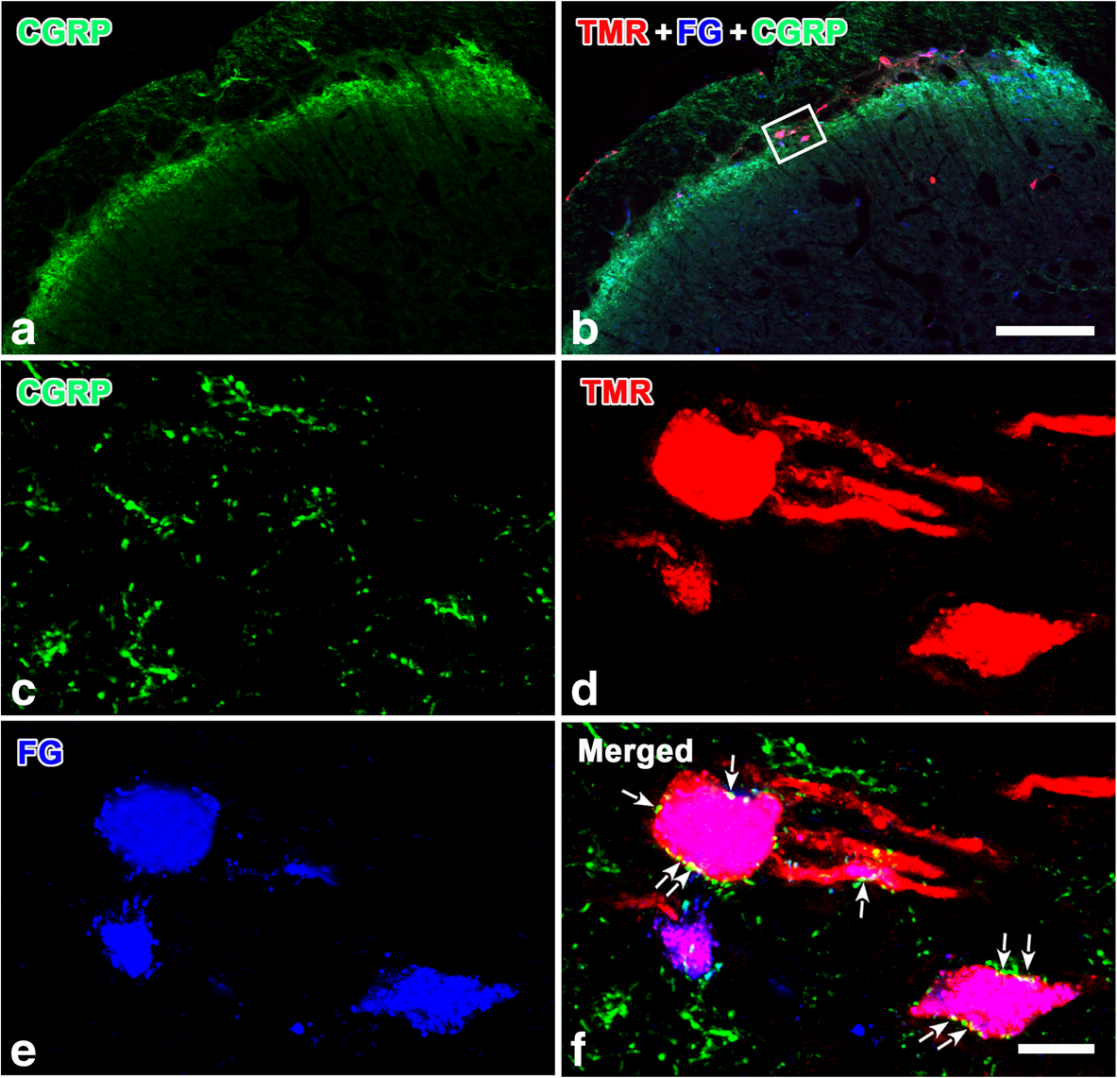
CGRP-positive terminals have close contacts with collateral projection neurons in the Vc superficial layer. TMR was injected into the right VPM and Po of the thalamus, and FG was injected into the left PBN in three rats (R4, R7, and R13, Fig. 2). TMR, FG and CGRP immunoreactivity in the superficial layer of the Vc are visualized with Alexa 594 (*red*), Alexa 647 (*blue*) and fluorescein (*green*), respectively. The boxed area in (**b**) is magnified in (**c-f**). The *arrows* in f indicate that some CGRP-LI axonal terminals are in close contact with TMR/FG double-labeled cell body or dendritic profiles. *Scale bar* 200 μm (in **a** and **b**); 20 μm (in **c**-**f**)

To further investigate whether the Vc collateral projection neurons previously identified directly received peripheral nociceptive afferents, a triple-labeled immunofluorescence histochemical technique for CGRP, TMR and FG was used. Under a confocal laser scanning microscope, CGRP-LI was mainly observed in axonal terminals in the superficial layers of the Vc (Fig. 9a, b). No CGRP-positive products were identified in the cell bodies or dendrite profiles. Some CGRP-LI axonal terminals were observed to be in close apposition with TMR/FG double-labeled neuronal cell bodies and dendrites in laminae I and II of the Vc (Fig. 9f).

The pre-embedded triple immune-electron microscopic method was performed in three rats to further examine whether CGRP-LI axon terminals may form synaptic contacts with the PBN and thalamus collateral projection neurons in the Vc. After the WGA-HRP injection into the right thalamus and the FG injection into the left PBN, retrograde tract tracing combined with CGRP-like immunohistochemistry was visualized with a typical TMB reaction method for WGA-HRP and the immunogold silver method for FG, whereas CGRP-labeling was identified by the immunoperoxidase method.

**Fig. 10:**
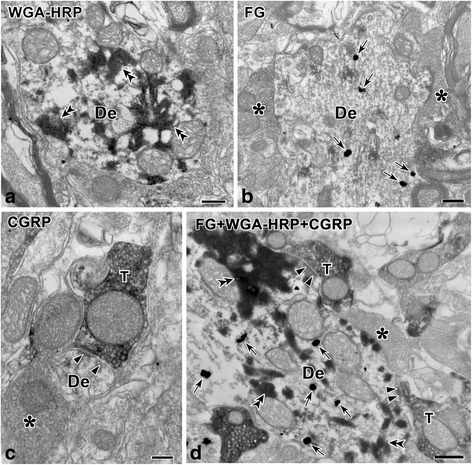
Electron microphotographs of the superficial layer of the Vc after triple-labeling with WGA-HRP, FG and CGRP. Axon terminals labeled with CGRP (**c**, **d**) form asymmetric synapses (*black arrowheads*) with WGA-HRP and FG dually labeled dendritic profiles (De) in the superficial layer of the Vc after a WGA-HRP injection into the right thalamus and an FG injection into the left LPB. CGRP-LI is represented with electron-dense, amorphous products of DAB reaction (**c**, **d**). Silver-intensified gold particles produced with the immunogold-silver method indicate FG labeling (**b**, **d**; *arrows*). Electron-dense, amorphous material produced by the peroxidase reaction indicate WGA-HRP labeling (TMB reaction; **a**, **d**; *double arrowheads*). *De*, dendritic profiles; *T,* CGRP-LI axon terminals; *, CGRP-negative axon terminals. *Scale bar* 250 nm (**a**, **b**, **c** and **d**)

In the Vc, electron-dense peroxidase reaction products (DAB reaction) that indicated CGRP labeling were identified within axon terminals, which were filled with round, black, clearly defined synaptic vesicles (Fig. 10c, d). In the superficial layer of the Vc, TMB reaction products that consisted of black crystals with high electronic densities indicating WGA-HRP labeling were identified in the cytoplasm and large dendritic processes of neurons that projected to the VPM and Po regions of the thalamus (Fig. 10a, d, double arrowheads). Some of the cytoplasm and large dendritic processes of neurons were also labeled with silver-intensified gold particles indicating FG labeling (Fig. 10b, d, arrows), which projected to the parabrachial region. Some of the neuronal cell bodies or dendrite profiles in the superficial layer of the Vc often showed WGA-HRP/FG double-labeling (Fig. 10d). Moreover, the CGRP-LI axon terminals were often observed in asymmetric synaptic contact with dendritic profiles that were labeled retrogradely with WGA-HRP/FG (Fig. 10d), which indicates that these neurons collaterally project to the thalamus and PBN.

## Discussion

It has been reported that some projection neurons in lamina I of the spinal dorsal horn [26] or medullary dorsal horn (Vc) of the rat [4] send their axons to both the thalamus and the PBN by way of axon collaterals; however, no previous study has reported the existence of these neurons in the Vo, Vi, and Vc or which VGLUTs may be expressed in the T-T and T-P collateral projection neurons in the Vsp. Thus, in the present study, retrograde tract tracing was performed to examine whether Vsp neurons may be labeled with two types of retrograde tracers that were injected into the thalamus and PBN of each rat. FISH histochemistry was further performed to examine the expression of VGLUT1 mRNA and VGLUT2 mRNA in these collaterally projecting neurons.

The results of the present study have indicated the existence of collaterally projecting neurons in the Vo, Vi and Vc, and these collateral projection neurons in the Vo, Vi and Vc exclusively expressed VGLUT2 mRNA and not VGLUT1 mRNA, with the exception of a few Vo collateral projection neurons that were VGLUT1 mRNA-positive. Moreover, the collaterally projecting neurons in the Vc may be related to the transmission of orofacial nociceptive information.

### VGLUT1 and VGLUT2 mRNA-expressing neurons in Vsp send axon collaterals to both the thalamus and the PBN

It has been reported that the trigeminothalamic fibers of the rat project to the thalamus bilaterally with contralateral predominance [27, 28]. Therefore, in the present study, TMR was injected into the contralateral thalamus, and it was ensured that the injection site covered the VPM and Po. TMR-labeled neurons were distributed in all subdivisions of the contralateral Vsp (Vo, Vi, and Vc). They were most prevalent in the Vi, followed by the Vo, with the smallest in number in the Vc. These data appear to be in fairly good accordance with data previously reported in the rat [15, 29]. The TMR-labeled neurons in the Vc showed a laminar pattern, with most of the retrogradely labeled neurons in the superficial layers (laminae I and II) as previously reported [4, 30]. Previous studies regarding direct projection from the Vo to the thalamus are contradictory: studies [29, 31] have shown retrogradely labeled neurons in the Vo, whereas other studies [2, 32] have not. In the present study, we observed TMR-labeled neurons throughout the Vo, with more neurons in the ventrolateral part. This distribution pattern is similar to that reported by Guy et al. [31].

It has been established that the projection fibers from the Vc to the PBN in the rat mainly terminate in the PBN portions lateral and ventromedial to the superior cerebellar peduncle [4, 33–35]. These PBN areas of termination of projection fibers from the ipsilateral Vsp (Vo, Vi, Vc) appeared to be included in the FG injection sites in the present study. As the PBN is dorsomedial to the Vp, the injection site was strictly limited within the PBN without contaminating the Vp, which receives direct projections from the Vsp. The FG-labeled neurons were observed in the bilateral Vsp with ipsilateral predominance. This finding is consistent with previous studies that described direct Vsp projections to the PBN with ipsilateral predominance in the rat [33, 35–37]. According to the present results, the PBN-projecting neurons were distributed among the entire Vo and Vi, whereas Vc neurons that send projections to the PBN were mainly located in the superficial layer, which is also consistent with previous studies [4, 34, 35, 38].

In the present study, following the TMR injection contralaterally into the thalamus and the FG injection ipsilaterally into the PBN, TMR/FG double-labeled neurons in the Vo, Vi and Vc were frequently encountered. Neurons dually labeled for TMR and FG were distributed in all subdivisions of the Vsp (Vo, Vi, and Vc); they were most prevalent in the Vi, followed by the Vo, with the smallest number in the Vc (Table 1). In the Vc, the TMR/FG double-labeled neurons were distributed in a laminar pattern, with the vast majority in lamina I. A previous study [4] also indicated that neurons in the Vc sent collateral projections to both the thalamus and the PBN, with more than 90% of the projecting neurons distributed in lamina I. Thus, the Vc neurons double-labeled with TMR/FG, which were observed in the present study, are considered to represent fairly well the Vc neurons that send their axons both ipsilaterally to the PBN and contralaterally to the thalamus by way of axon collaterals. Moreover, the results of the present study have further indicated the existence of neurons in the Vi and Vo that also send collateral projection to both the PBN and the thalamus; however, these collateral projection neurons have not been reported in previous studies.

In the present study, FISH histochemistry for VGLUT1 or VGLUT2 mRNA was combined with double retrograde tract tracing using TMR injected into the thalamus and FG injected into the PBN. The results indicated that most TMR/FG doubly labeled Vsp neurons express VGLUT2 mRNA (90.3% in the Vo, 93.0% in the Vi, and 85.4% in the Vc), whereas almost no TMR/FG-labeled Vi and Vc neurons express VGLUT1 mRNA (Tables 1 and 2). Dual FISH histochemistry performed in a previous study [16] also indicated that no single Vsp neurons co-express VGLUT1 and VGLUT2 mRNAs. Thus, it was assumed that glutamatergic T-T and T-P collateral projection neurons in the Vsp mainly express VGLUT2 mRNA.

### Collateral projection neurons in Vc are related to the transmission of orofacial nociceptive information

The Vc is often referred to as the medullary dorsal horn and is considered to be homologous to the spinal dorsal horn both structurally and functionally [39]. The superficial laminae of the Vc contains many neurons that constitute the main relay for nociceptive primary afferents from the orofacial regions [39–41], and previous studies have indicated that substance P (SP)-containing small-diameter primary afferent fibers and CGRP-LI primary afferent fibers terminate mainly in laminae I and II [42–45]. Thus, in the present study, when some TMR/FG double-labeled soma and dendrites of neurons in laminae I and II of the Vc were in apposition to CGRP-LI terminals under the confocal laser-scanning microscope, they were assumed to receive and transmit nociceptive inputs. Furthermore, the results of the electron microscopy also indicated that CGRP-LI axon terminals (labeled with DAB reaction) formed asymmetric synapses with the soma or dendrite profiles of the TMR/FG double-labeled collateral projection neurons in the Vc. In accordance with the present data, previous studies in primates have indicated that many nociceptive neurons in the Vc receive direct primary nociceptive afferent input and send projection fibers to both the PBN and the thalamus, including the VPM and Po, by way of axon collaterals [4, 26, 46–48].

Furthermore, the nociceptive nature of Vc neurons that were retrogradely labeled with TMR and FG injected into the thalamus and PBN was identified by the expression of Fos-LI after the injection of formalin solution into the upper lip of rats. Previous studies have shown that noxious stimulation of different orofacial sites induced Fos-LI in the ipsilateral Vc, mainly in laminae I and II [18, 49–52]. Therefore, Fos-LI has often been used for the investigation of somatotopy in the trigeminal nociceptive pathways [18, 53, 54]. In the present study, after subcutaneous injection of formalin into the upper lip of rats, Fos-LI was induced in many neurons in the Vc, particularly in the superficial laminae of the dorsomedial part. This distribution pattern of Fos-LI was consistent with previous studies [18, 19, 55, 56] and appeared to be compatible with the somatotopy of the projection of the trigeminal nerve [24]. We also performed triple-labeling of TMR/FG/Fos to identify the expression of Fos-LI in TMR/FG double-labeled neurons. The neurons that supply relatively localized orofacial regions are somatotopically organized within the Vc [24]. We noxiously stimulated only the upper lip, and it may be assumed that only some of the nociceptive neurons in the Vc were activated. Thus, the present result that 34.0% of TMR/FG double-labeled neurons expressed Fos-LI may indicate that a substantial proportion of the collateral projection neurons in the Vc are activated by noxious stimulation of the orofacial region.

### Functional implications of the glutamatergic collateral projection neurons in Vsp

The thalamus and PBN are two nuclei with distinctly different roles. As shown in the present results, the injection site in the thalamus covered the VPM and Po. These two thalamic relay nuclei receive their afferent input from the Vsp and project to the somatosensory cortex in a complementary pattern [15, 16, 57, 58]. Both nuclei are critical in the formation of discriminative somatic sensation of orofacial regions [39, 59, 60]. The function of the PBN is relatively complicated. It is involved not only in gustatory functions [61–63] and autonomic regulatory processes [38, 64–68] but also affective processes [69–71], particularly in the affective aspects of pain [5, 38, 72, 73]. The information conveyed by the T-P pathways likely contributes more to the affective component of the pain experience than to the discriminative somatic sensation [39, 74]. In brief, the T-T and T-P projections are related to the discriminative somatic sensation and the affective component of orofacial sensation, respectively.

*In situ* hybridization detection was used for VGLUT1 and VGLUT2 mRNA, which have been well established as the specific markers for glutamatergic neurons in the CNS [10–14, 75–78]. The distribution of VGLUT1 and VGLUT2 in the CNS exhibited a complementary pattern [14], which indicates that there may be a distinct functional difference between these two proteins. In the Vsp, as shown in a previous study, the distribution and projection of VGLUT1 and VGLUT2 mRNA-positive neurons are also different [17]. VGLUT1 mRNA-expressing neurons are distributed in the Vo, Vi, and Vc, and some VGLUT1 mRNA-expressing neurons in the Vi, and a few neurons in the Vo send VGLUT1-positive axon terminals to the trigeminal motor nucleus [17] and cerebellum [15]. Moreover, nearly all Vo, Vi and Vc neurons express VGLUT2 mRNA signals and send VGLUT2-positive axon terminals to the contralateral thalamus [15]. The current findings showed that Vsp neurons sending collateral projections to the thalamus and PBN mainly expressed VGLUT2 mRNA signals, which indicates that VGLUT2 and not VGLUT1 was potentially involved in the transmission of orofacial information from the Vsp to the thalamus and PBN.

The subnuclei of the Vsp (Vo, Vi, and Vc) have been indicated to be different in their functional properties: they are related to different types of sensory information from orofacial regions [24]. Among these nuclei, the Vc has been widely investigated for its role in the transmission of orofacial nociceptive information. According to the results of the present and previous studies, Vc neurons sending collateral projections to the thalamus and PBN were mainly distributed in the superficial laminae [4, 79]. The superficial laminae of the Vc are the primary gateway for the peripheral noxious information transmitted from the orofacial region [39, 80, 81]. CGRP is regarded as a key mediator released from trigeminal ganglion neurons to the superficial laminae of the Vc after the stimulation of sensory nerve endings and is responsible for the transmission of pain sensation [82]. In the present study, we determined that CGRP-LI terminals formed asymmetric synapses with somatic or dendritic profiles of the neurons projecting collaterally to the thalamus and PBN in the superficial laminae of the Vc. The collateral projecting neurons were also activated after painful stimulation of the lip. It may be assumed from the present results that the VGLUT2-positive neurons in the superficial laminae of the Vc may receive pain information from the primary afferent fibers of the trigeminal nerve and simultaneously relay it to the thalamus and PBN.

According to the present data, the VGLUT2-expressing neurons in the Vi and Vo also send axonal collaterals to the thalamus and PBN. Previous studies have shown that the Vi is primarily concerned with the transmission of tactile sensation and low-threshold signals from orofacial regions, and Vi neurons predominately respond to the stimulation of individual vibrissae in rats [83, 84]. Vo neurons are closely related to the transmission of orofacial proprioceptive sensation [24]. Thus, it is plausible that VGLUT2-expressing neurons in the Vi or Vo may relay orofacial tactile sensation or proprioceptive sensation, respectively, to the thalamus and PBN. The T-T projection from the Vi is related to discriminative orofacial tactile sensation from the vibrissae [24]. The T-P projection from the Vi may be related to affective reactions evoked by orofacial tactile sensation from the vibrissae. Moreover, it has been reported that amputation of vibrissae may induce anxiety in rats [85]. The T-T and T-P projections from the Vo are mainly concerned with discriminative orofacial proprioceptive sensation and concomitant affective reactions, respectively [24]. Whether the discriminative sensation and affective reactions of orofacial tactile and proprioceptive sensation are transmitted by these collateral projection neurons in the Vi and Vo remains to be discovered. Our data further confirmed that trigemino-thalamic and trigemino-parabrachial collateral projection neurons were VGLUT2 mRNA-positive, which further indicates that orofacial nociceptive information uses glutamate as a neurotransmitter recruited by VGLUT2.

## Conclusions

In summary, the present study indicates T-T and T-P collateral projection neurons that send their axon terminals to both the thalamus and the PBN by way of axon collaterals are mainly localized in the Vo, Vi and Vc. Glutamatergic T-T and T-P collateral projection neurons in the Vo, Vi and Vc mainly express VGLUT2 mRNA. Some T-T and T-P collateral projection neurons that express Fos-LI in the Vc are closely related to the transmission of orofacial nociceptive information after subcutaneous injection of formalin into the upper lip. Moreover, the collateral projection neurons in the Vc that formed synaptic contacts with CGRP-LI primary afferent axon terminals were also considered nociceptive.

## Abbreviations

3 V: Third ventricle
4 V: Fourth ventricle
CM: Central medial thalamic nucleus
cp: Cerebral peduncle
f: Fornix
FG: Fluoro-Gold
FISH: Fluorescence in situ hybridization
fr: Fasciculus retroflexus
Hb: Habenular nucleus
ic: Internal capsule
KF: Kölliker-Fuse nucleus
LC: Locus coeruleus
LD: Laterodorsal thalamic nucleus
LPB: Lateral parabrachial nucleus
MD: Mediodorsal thalamic nucleus
MPB: Medial parabrachial nucleus
mt: Mammillothalamic tract
opt: Optic tract
PAG: Periaqueductal gray
PBN: Parabrachial nucleus
PF: Parafascicular thalamic nucleus
Po: Posterior thalamic nuclear group
Rt: Reticular thalamic nucleus
scp: Superior cerebellar peduncle
TMR: Tetramethylrhodamine
T-P: Trigemino-parabrachial (T-P) pathways
T-T: Trigemino-thalamic pathways
Vc: Caudal subnucleus of spinal trigeminal nucleus
VGLUT: Vesicular glutamate transporter
Vi: Interpolar subnucleus of spinal trigeminal nucleus
Vmes: Trigeminal mesencephalic nucleus
Vo: Oral subnucleus of spinal trigeminal nucleus
Vp: Principal sensory trigeminal nucleus
VPL: Ventral posterolateral thalamic nucleus
VPM: Ventral posteromedial thalamic nucleus
Vsp: Spinal trigeminal nucleus
ZI: Zona incerta
